# Nanopore long-read RNA sequencing reveals functional alternative splicing variants in human vascular smooth muscle cells

**DOI:** 10.1038/s42003-023-05481-y

**Published:** 2023-10-31

**Authors:** Hao Wu, Yicheng Lu, Zhenzhen Duan, Jingni Wu, Minghui Lin, Yangjun Wu, Siyang Han, Tongqi Li, Yuqi Fan, Xiaoyuan Hu, Hongyan Xiao, Jiaxuan Feng, Zhiqian Lu, Deping Kong, Shengli Li

**Affiliations:** 1 Department of Cardiovascular Surgery, Shanghai Sixth People’s Hospital Affiliated to Shanghai Jiao Tong University School of Medicine, Shanghai, China; 2 Precision Research Center for Refractory Diseases, Institute for Clinical Research, Shanghai General Hospital, Shanghai Jiao Tong University School of Medicine, Shanghai, China; 3 Department of Gynecological Oncology, Fudan University Shanghai Cancer Center, Shanghai, China; 4 Department of Ophthalmology, Shanghai General Hospital, Shanghai Jiao Tong University School of Medicine, Shanghai, China; 5 North Cross School Shanghai, Shanghai, China; 6 H. Milton Stewart School of Industrial and Systems Engineering, College of Engineering, Geogia Institute of Technology, Atlanta, GA USA; 7 Department of Cardiac Surgery, Wuhan Asia Heart Hospital, Wuhan University of Science and Technology, Wuhan, China; 8 Department of Vascular Surgery and Intervention Center, Shanghai General Hospital, Shanghai Jiao Tong University School of Medicine, Shanghai, China

**Keywords:** Data processing, High-throughput screening, Cardiac regeneration

## Abstract

Vascular smooth muscle cells (VSMCs) are the major contributor to vascular repair and remodeling, which showed high level of phenotypic plasticity. Abnormalities in VSMC plasticity can lead to multiple cardiovascular diseases, wherein alternative splicing plays important roles. However, alternative splicing variants in VSMC plasticity are not fully understood. Here we systematically characterized the long-read transcriptome and their dysregulation in  human aortic smooth muscle cells (HASMCs) by employing the Oxford Nanopore Technologies long-read RNA sequencing in HASMCs that are separately treated with platelet-derived growth factor, transforming growth factor, and hsa-miR-221-3P transfection. Our analysis reveals frequent alternative splicing events and thousands of unannotated transcripts generated from alternative splicing. HASMCs treated with different factors exhibit distinct transcriptional reprogramming modulated by alternative splicing. We also found that unannotated transcripts produce different open reading frames compared to the annotated transcripts. Finally, we experimentally validated the unannotated transcript derived from gene *CISD1*, namely *CISD1-u*, which plays a role in the phenotypic switch of HASMCs. Our study characterizes the phenotypic modulation of HASMCs from an insight of long-read transcriptome, which would promote the understanding and the manipulation of HASMC plasticity in cardiovascular diseases.

## Abstract (web summary)

Long-read RNA-seq analysis identifies and measures novel transcripts in cultured human aortic smooth muscle cells under basal conditions and after treatment with cytokines or CISD1-u knockdown.

## Introduction

Vascular smooth muscle cells (VSMCs) are located within the vasculature and constitute the major cells of the medial layer of arteries^1,2^. VSMCs participate in arterial contraction and extracellular matrix (ECM) production, which are highly differentiated in healthy vessels with fully functional phenotypes. The phenotypic modulation of VSMCs from a state to another, such as from differentiated to dedifferentiated state, has been shown to play important roles in various cardiovascular diseases, such as atherosclerosis and restenosis^3^, hypertension^4^, and other aging-related diseases^5^. Two major phenotypes of VSMCs are contractile and non-contractile (synthetic), wherein contractile phenotype, including quiescent and differentiated VSMCs, mainly modulate the size reduction or shortening of muscles, while the synthetic phenotype, which is more likely to migrate or proliferate, contributes to the vascular remodeling under various pathophysiological conditions^6^. A variety of factors could induce phenotypic changes in VSMCs. The platelet-derived growth factor (PDGF), which is a potent mitogen, makes a major contribution to the phenotypic switch from contractile to proliferative state of VSMCs^7,8^, is characterized by high expression of constriction genes, such as osteopontin (*OPN*), kruppel like factor 4 (*KLF4*), *KLF5*, and Cyclin D1 (*CCND1*)^9^. The transforming growth factor beta (TGFβ) has been demonstrated to enhance the proliferation^10^ and maintain the differentiation state^11^ of VSMCs, wherein contractile markers include smooth muscle α-actin (SMαA), smooth muscle 22α (SM22α), smooth muscle calponin (CNN1), smooth muscle myosin heavy chain (SM-myh11), and smoothelin-B (SMTN-B), and transgelin (TAGLN)^12^. Growing evidence has shown that microRNAs (such as miR-145^13^, miR-221^14^, miR-124^15^, and miR-22^16^) play an important role in regulating VSMC phenotypic modulation. These are all suitable factors for the treatment of VSMCs to establish good models of phenotypic changes.

Aberrant alternative splicing has been demonstrated to play crucial roles in human complex diseases, including cancer^17,18^ and cardiovascular diseases^19,20^. Different transcripts that might perform distinct functions could be produced from the same genes through alternative splicing^21–23^. The extensive applications of high-throughput RNA sequencing (RNA-seq) technologies have accelerated the discovery of alternative splicing events that are frequently dysregulated in VSMCs^24,25^. Eric et al. found that the Myocd_v3 transcript could enhance the contractility and decrease the proliferation of VSMCs, while the other transcript derived from the gene Myocd, Myocd_v1, inhibited cellular contractility and induced modest proliferation^26^. They also revealed that the Myocd_v3/Myocd_v1 transcript balance could be modulated by the RNA binding protein QKI through regulating the alternative splicing of gene Myocd. Although studies based on short RNA-seq data have largely promoted our understanding of the roles of alternative splicing in cardiovascular diseases, most alternative splicing identification is based on computational inference of transcript structures from short RNA-seq reads. The rapid development of long-read RNA-seq provides an opportunity to evaluate full-length transcripts and more accurate identification of alternative splicing events^27,28^. However, the study of transcriptomics based on long-read RNA-seq data is scarce in VSMCs. In the present study, we performed Oxford Nanopore Technologies (ONT) long-read RNA-seq in three human aortic smooth muscle cell (HASMC) groups with the treatment of PDGF, TGFβ, and miR-221, respectively. We presented a landscape of full-length transcripts in human VSMCs, and compared the transcriptome dysregulation in different sample groups. We further characterized alternative splicing events in HASMCs with different treatments, and identified numbers of unannotated transcripts generated from alternative splicing. We experimentally validated the expression of an unannotated transcript that was derived from the *CISD1* gene, namely *CISD1-u*, and its function in the phenotypic modulation of HASMCs.

## Results

### The landscape of long-read transcriptome in HASMCs

**Fig. 1:**
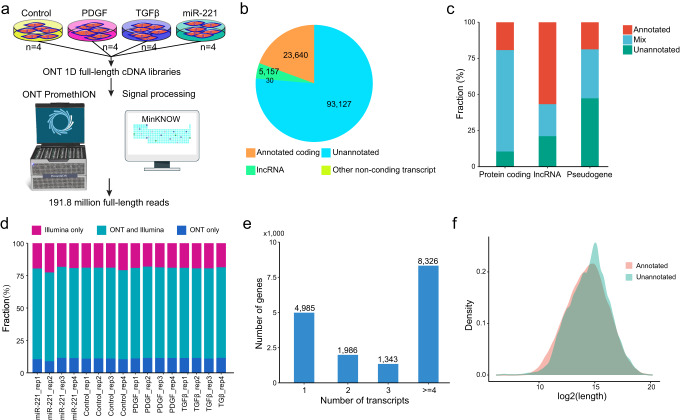
Long-read nanopore RNA sequencing to identify full-length transcripts in VSMCs. **a** Schematic of experimental design for this study. Primary HASMCs (passage 3 to 6) were transfected with negative control miRNA or hsa-miR-221-3P (50 nM) for 36 h. Subsequently, cells were treated with vehicle control, PDGF (15 ng/mL), or TGFβ (10 ng/ml) for another 24 h and analyzed. *n* = 4 for each group. **b** Composition of different transcript types in all detected full-length transcripts. **c** Proportion of genes with annotated and unannotated transcripts in different gene types. **d** Stack plot shows the fractions of genes detected in different sequencing technologies in each sample. **e** Bar plot shows the number of transcripts in genes detected in ONT platform. **f** Length distribution of annotated and unannotated transcripts detected in ONT platform.

To investigate the landscape of long-read transcriptome in HASMCs, we conducted ONT 1D cDNA sequencing in 16 HASMC samples (Fig. 1a). These samples were derived from control, PDGF-cultured, TGFβ-cultured, and hsa-miR-221-3P-transfected human VSMCs, with four replicate samples in each group. Appropriately 191.8 million long-read reads were obtained from all samples with an average passing percentage of 97.51% (Supplementary Table 1 and Table 2). The median read length ranged from 1281 to 1555 nt. Of these passing reads, an average of 76.83% were mapped to the human reference genome. In average, 13,387 genes and 253,174 transcripts were detected in each sample. ONT long-read RNA-seq transcript quantification showed high correlation with the Illumina short-read RNA-seq data across different samples (Pearson *r* range is 0.77–0.88), indicating that our ONT long-read RNA-seq data recapitulates those in Illumina sequencing of HASMC samples (Supplementary Fig. 1a). A total of 121,954 transcripts were detected in all samples (Fig. 1b). The majority of these transcripts (76.36% of all detected transcripts) were unannotated. Most of the protein coding genes were found to express both annotated and unannotated transcripts (Fig. 1c). Meanwhile, unannotated transcripts were detected in a considerable fraction of genes, especially in pseudogenes. Comparing genes detected by ONT and Illumina RNA-seq data exhibited that the majority were identified by both sequencing technologies (Fig. 1d). However, in the transcript level, most were specifically detected by ONT or Illumina RNA-seq data (Supplementary Fig. 1b). Transcripts that were detected by both ONT and Illumina sequencing showed significantly higher expression levels than those in only long-read or short-read RNA-seq data (Supplementary Fig. 1c). About half of detected genes (*n* = 8326) were found to express no less than four different transcripts, suggesting that most genes might undergo alternative splicing (Fig. 1e). The unannotated transcripts showed a relatively higher fraction of transcripts with longer length compared to annotated transcripts, which had the highest density of length distribution at appropriately 32,768 nt (Fig. 1f). Transcripts that were detected only by ONT long-read RNA-seq were much longer (Supplementary Fig. 1d). These results indicate that ONT long-read and Illumina short-read RNA-seq showed different performance in detecting lowly expressed and long transcripts. In addition, over 50 percent of known protein coding genes were detected in HASMCs by both ONT and Illumina sequencing (Supplementary Fig. 1e). But ONT showed relatively lower ability to detect noncoding RNAs than Illumina short-read sequencing in our dataset. In summary, our analysis presented a systematic characterization of long-read transcriptome in HASMCs, and compared to those screened by Illumina short-read sequencing.

### Dysregulation of long-read transcriptome in miR-221-transfected, PDGF-cultured, and TGFβ-cultured HASMCs

**Fig. 2:**
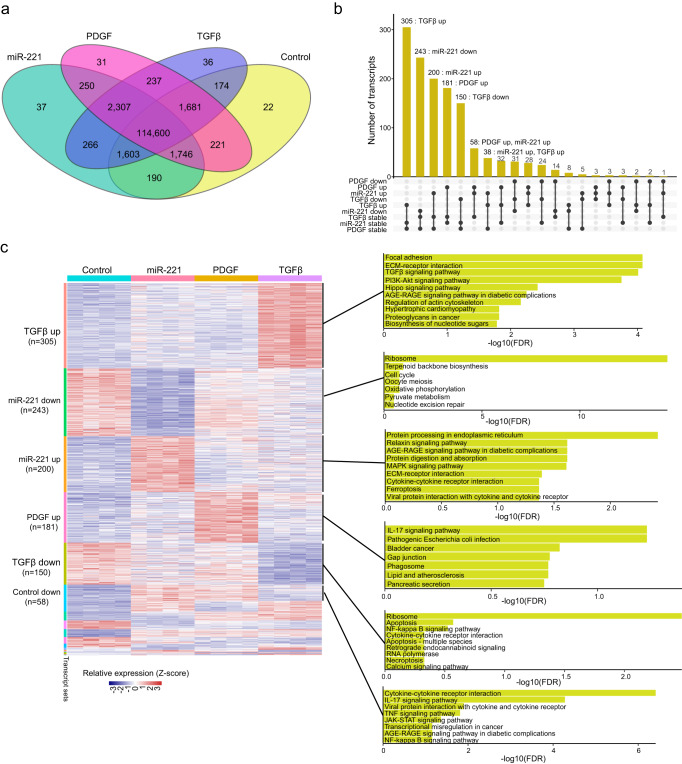
Differential transcripts in the miR-221, PDGF, and TGFβ groups. **a** Venn plot shows overlaps of detected transcripts among different sample groups. **b** Upset plot shows the numbers of shared transcripts that were dysregulated in different groups. **c** Heatmap shows the relative expression levels of differential transcripts across all samples. Transcripts are ordered by sets in **b**. Numbers on the left indicate the number of differential transcripts. Bar plots on the right show the significantly enriched pathways for each set of transcripts.

We further examined the differentially expressed transcripts (DETs) among distinct HASMC groups. The vast majority of transcripts (114,600 transcripts, 94.0% of all) were detected in all four HASMC sample groups (Fig. 2a). DETs were then identified by separately comparing miR-221, PDGF, and TGFβ groups with the control group. Thousands of transcripts were found to be dysregulated in individual groups, including 692 unannotated transcripts (Supplementary Data 1). In particular, 336 upregulated and 423 downregulated transcripts were identified in the miR-221 group (Supplementary Fig. 2a). The PDGF group showed 305 upregulated and 203 downregulated transcripts (Supplementary Fig. 2c). HASMCs cultured with TGFβ exhibited 418 upregulated and 341 downregulated transcripts compared to the control HASMCs (Supplementary Fig. 2e). The enrichment of DETs showed biological processes associated with the phenotypic switch of HASMCs (Supplementary Fig. 2b, d, f, and Supplementary Data 2). For example, in TGFβ-treated HASMCS, apart from the typical “TGFβ signaling pathway”, the upregulated “Hippo signaling pathway” is well known as a central regulator of phenotypic switch of vascular smooth cells (VSMCs)^29^, linked to various fibrotic diseases and characterized with excessive extracellular matrix accumulation^30^. Especially, YAP activation facilitates the gene expression of contractile marker smooth muscle α-actin (SMαA)^31^; For PDGF-treatment, highly expressed genes were enriched in “TNF signaling pathway” and “cytokine-cytokine receptor interaction”, prone to an inflammatory status. Single-cell sequencing and lineage-tracing strategy have confirmed the dedifferentiated state of VSMCs are associated with macrophage-like VSMCs, characterized with macrophage surface markers and functions^32,33^; miR-221, a downstream target of PGDF, shared most similar differential transcripts with PGDF treatment, enriched in “cytokine-cytokine receptor interaction” and “TNF signaling pathway”, highly associated with constriction phenotype. About 73% (305 in 418) of upregulated and 44% (150 in 341) of downregulated transcripts in the TGFβ group showed no expression changes in other groups (Fig. 2b). The transcriptional variations of these transcripts might be exclusively induced by TGFβ. In HASMCs with miR-221 transfection, about 57 % (243 in 423) of downregulated and 60% (200 in 336) of upregulated transcripts were specifically modulated by miR-221. In PDGF-cultured HASMCs, about 59% (181 in 305) of upregulated transcripts exhibited exclusive upregulation, while all downregulated transcripts were found upregulated or downregulated in the miR-221 or TGFβ group. Transcripts that showed specific upregulation in the TGFβ group were enriched in “focal adhesion”, “ECM-receptor interaction”, “TGFβ signaling pathway”, and “PI3K-Akt signaling pathway” (Fig. 2c, and Supplementary Data 3 and Data 4). Highly expressed transcripts in the miR-221 group were significantly enriched in “protein processing in endoplasmic reticulum” and “relaxin signaling pathway”. The “IL-17 signaling pathway” was highly enriched in the PDGF group. In conclusion, miR-221, PDGF, and TGFβ induced specific transcriptome variations in HASMCs.

### Alternative splicing events are frequently identified in HASMCs by long-read RNA sequencing

**Fig. 3:**
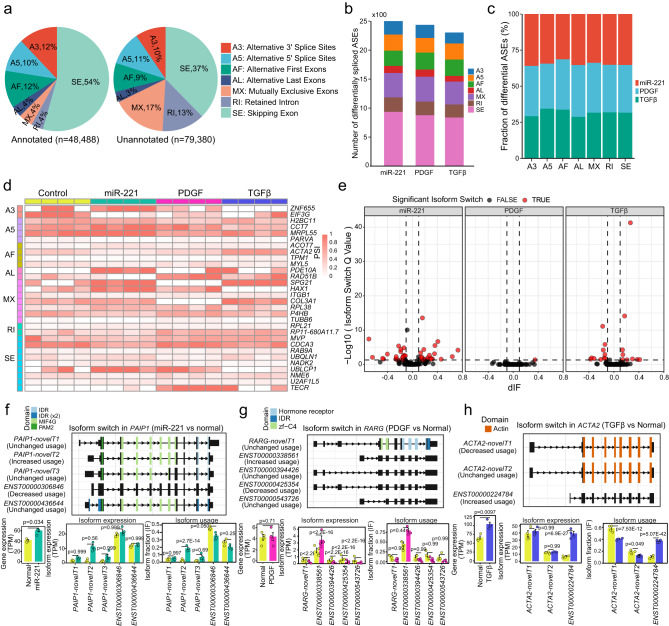
Aberrant alternative splicing events in VSMCs. **a** The percentages of different ASE types in annotated and unannotated genes. **b** The number of differential ASEs in each sample group. **c** The percentages of differential ASEs for each ASE type across different sample groups. **d** Heatmap shows PSI values of representative genes that were detected with top differential ASEs across samples. **e** Volcano plots show the difference of switching isoforms in miR-221, PDGF, and TGFβ groups. **f** The isoform structures, gene expression, isoform expression, and isoform usages of the *PAIP1* gene in the miR-221 group. **g** The isoform switching details of the *RARG* gene in the PDGF group. **h** The isoform switching details of the *ACTA2* gene in the TGFβ group. Error bars represent the means ± SDs. In (f-h), differential analysis of gene and isoform expression were performed by DESeq2 package, and isoform usage was by DEXSeq package. *n* = 4 independent samples were included in each group. Abbreviations in f-h: IDR, intrinsically disordered region; MIF4G, middle domain of eukaryotic initiation factor 4G; PAM2, PABP-interacting motif 2; zf-C4, zinc finger C4 type.

As multiple transcripts were detected in most genes, alternative splicing events (ASEs) were supposed to frequently occur in HASMCs. Next, we identified ASEs in all detected genes to explore the transcriptional diversity of HASMCs. ASEs were identified by using SUPPA2 (version 2.3) from the transcript expression matrix and gene annotation (see Methods). In total, 48,488 and 79,380 ASEs were identified in annotated and unannotated genes, respectively (Fig. 3a and Supplementary Data 5). Seven different ASE types were identified, including skipped exon (SE), mutually exclusive exon (MX), alternative 5’ splice site (A5), alternative 3’ splice site (A3), retained intron (RI), alternative first exon (AF), and alternative last exon (AL). SE was the most frequent splicing type in both annotated and unannotated genes. AF and A3 were the second most frequent ASEs in annotated genes, while MX was the second most frequent splicing type in unannotated genes. Notably, intron retention constituted a considerable part (13%) in unannotated genes. Differential ASE analysis identified 2,502, 2,437 and 2,304 differentially spliced ASEs in the miR-221, PDGF, and TGFβ group, respectively (Fig. 3b). For each ASE type, the portion of differentially spliced ASEs were approximately the same across different sample groups (Fig. 3c). Most genes with differentially spliced ASEs were enriched in RNA metabolism-related processes (Supplementary Fig. 3). Some genes showed specific splicing patterns among different sample groups, which might be induced by specific stimulants (Fig. 3d). For example, the SE splicing of *TECR* gene was specifically found in HASMCs treated with PDGF. To investigate the splicing patterns that may cause substantial functional consequences, we performed genome-wide detection of significantly switching isoforms by using the IsoformSwitchAnalyzeR package (version 1.17.04) (see Methods). Changes of isoform usage were computed in each group, and IF values were calculated to evaluate the significance of isoform switching events. Compared to the control samples, we identified 41, 1, and 19 significant isoform switching events in the miR-221, PDGF, and TGFβ samples, respectively (Fig. 3e and Supplementary Data 6). In the miR-221 group, the *PAIP1* gene showed differential expression, but only a newly identified transcript *PAIP1-novelT2* exhibited upregulated usage (Fig. 3f). The *RARG* gene showed no significant expression change at gene level, while multiple transcripts of *RARG* were differentially expressed in the PDGF HASMCs, wherein some were upregulated and some others were downregulated (Fig. 3g). In particular, the *ENST00000338561* transcript was significantly upregulated, while other transcripts were downregulated in the PDGF-treated HASMCs. The upregulation of transcript *ENST00000338561* was not identified in the gene-level analysis. Gene *ACTA2* was significantly upregulated in the TGFβ-treated VSMCs, whereas only the *ENST00000224784* transcript was upregulated (Fig. 3h). In conclusion, alternative exon usage analysis revealed more specific dysregulation of transcripts in HASMCs that might be overlooked in gene-level analysis.

### Unannotated transcripts generate different open reading frames

**Fig. 4:**
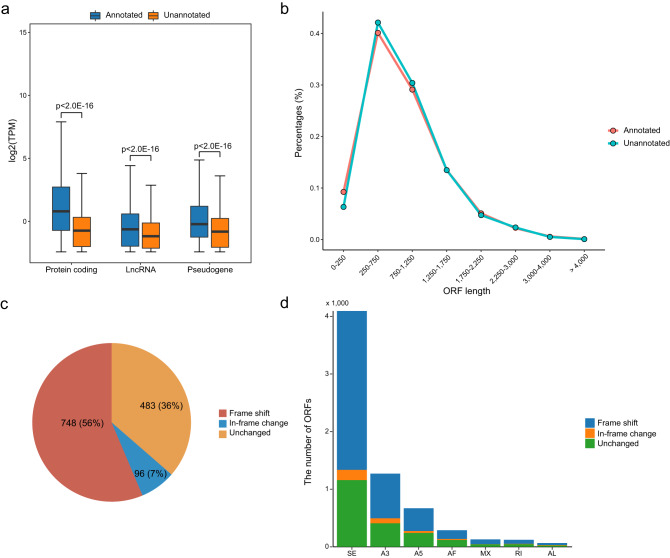
Expression levels and ORF changes in unannotated transcripts. **a** Box plots comparing the expression levels between annotated and unannotated transcripts in protein coding genes, lncRNAs, and pseudogenes. Each box represents the IQR and median of TPM value of each transcript, whiskers indicate 1.5 times the IQR. P, Wilcoxon’s rank-sum test. *n* = 443,200 annotated and *n* = 1,373,392 protein coding transcripts, *n* = 41,168 annotated and *n* = 39,984 unannotated lncRNA transcripts, *n* = 5472 annotated and *n* = 15,840 unannotated pseudogene transcripts, respectively. The individual expression values (data points) could be found in the Supplementary Data 8. **b** The length distributions of ORF from annotated and unannotated transcripts. **c** The percentage of transcripts with different ORF changes. **d** Bar plots showing the number of different ORF changes in each ASE type.

To further characterize the feature of unannotated transcripts, we compared the expression and open reading frames (ORFs) between annotated and unannotated transcripts. Overall, unannotated transcripts showed significantly low expression than annotated transcripts in all gene types (Fig. 4a), especially in top high expression level (expression rank 0–25%) (Supplementary Fig. 4a). In low expression levels (expression rank 75–100%), unannotated transcripts exhibited almost the same level as annotated transcripts (Supplementary Fig. 4a). To evaluate the effects of unannotated transcripts on gene translation, we predicted ORFs of annotated and unannotated transcripts by using the NCBI ORFfinder^34^. The ORF length distribution of annotated and unannotated transcripts was similar (Fig. 4b), which was consistent with previous studies^35^. Most ORFs (56%) of unannotated transcripts changed by frameshift compared to those of the annotated transcripts in the same genes (Fig. 4c). Approximately one-third of ORFs were unchanged in the unannotated transcripts. This observation was almost the same in different sample groups (Supplementary Fig. 4b–d). The SE alternative splicing events exhibited the largest number of ORF changes, followed by the A3 events (Fig. 4d). Our analysis revealed that newly identified unannotated transcripts had different ORFs that might impact the translation of host genes.

### The unannotated transcript *CISD1-u* plays a role in HASMC phenotypic switching

**Fig. 5:**
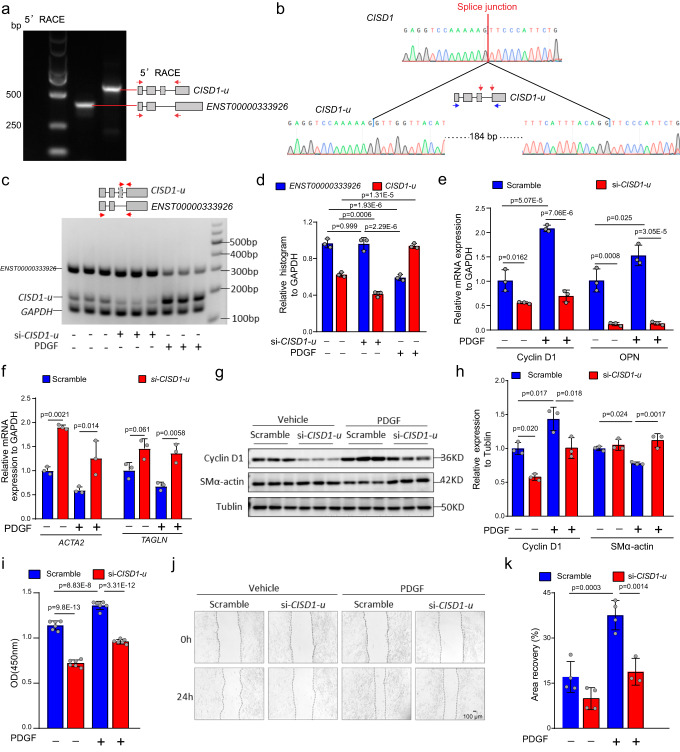
Experimental validation of the unannotated transcript *CISD1-u* derived from the *CISD1* gene. **a** The validation of the unannotated transcript *CISD1-u* in HASMCs by 5’ RACE. Red arrows indicate the primer pairs complementary to the indicated *CISD1-u* novel exon-intron boundary sequences used for 5’ RACE. **b** The sequence validation of the unannotated transcript *CISD1-u* in HASMCs by Sanger sequencing. Red arrows indicate the splice junction sites, and blue arrows indicate where the primers target. **c**, **d** Validation of the splicing in novel transcript *CISD1-u* by percent splicing inclusion **c** and quantification analysis **d** upon the treatment of si-*CISD1-u* or PDGF. Red arrows in **c** indicate the primer pairs used for semiquantitative RT-PCR. *n* = 3 independent samples were used in each group. **e** Expression quantification of cell proliferation-related genes (*Cyclin D1* and *OPN*) upon *CISD1-u* knockdown. *n* = 3 independent samples were used in each group. **f** Expression quantification of contraction-related genes (*ACTA2* and *TAGLN*) upon *CISD1-u* knockdown. *n* = 3 independent samples were used in each group. **g**, **h** Representative western blot and quantification of the protein levels of Cyclin D1 and SMα-actin upon *CISD1-u* knockdown post PDGF treatment. These western blots were derived from different runs with the same sample loading. GAPDH was used as a loading control. In **e**–**h**, primary HASMCs were transfected with scramble or si-*CISD1-u* (50 nM) for 36 h. Subsequently, cells were treated with vehicle control or PDGF (15 ng/ml) for another 24 h for mRNAs or 48 h for protein assays. *n* = 3 independent samples were used in each group. **i** Quantitative analysis of cell proliferation assay. *n* = 6 independent samples were used in each group. **j** Representative images of wound-healing assay. Images were taken at 0 and 24 h after scratching (white lines indicate wound edges). **k** Quantitative analysis wound-healing assay in **j**. Three or four independent samples were used in each group. In **i**–**k**, primary HASMCs were transfected with scramble or si-*CISD1-u* (50 nM). Cells were treated with vehicle control or PDGF (15 ng/ml) for another 24 h and subject to CCK8 cell proliferation assay or scratch-wound assay. Error bars represent the means ± SDs. *P:* two-way ANOVA with Bonferroni post hoc analysis.

To further investigate unannotated transcripts identified by ONT long-read RNA-seq, we experimentally validated the unannotated transcripts with top expression changes detected in the TGFβ (*FOXS1*, *CACNA2D3*, *ADAM19*, *UN41*, *MACF1*, and *MAP2*) (Supplementary Fig. 5a), PDGF (*CISD1*, *CACNA2D3*, *ADAM19*, and *RFX8*) (Supplementary Fig. 5b), and miR-221 (*TCIM*, *HSPH1*, *MDM2*, *ITM2B*, and *FAM114A1*) (Supplementary Fig. 5c) groups. The unannotated transcripts derived from gene *FOXS1*, *CACNA2D3*, *ADAM19*, *UN41*, *CISD1*, *ADAM19*, *HSPH1*, and *MDM2* showed upregulation in the qRT-PCR assays. Moreover, most of these novel transcripts could be identified in control human vascular tissues, as well as the aortic dissection (AD) tissue (Supplementary Fig. 5d), which underwent significant pathological phenotypical switch of VSMC (from contractile phenotype to synthetic phenotype) during AD development and progression^36^. Of note, the expression level of novel transcript from gene *CISD1*, referred as *CISD1-u* was consistently elevated in AD VSMC. The *CISD1* (CDGSH iron sulfur domain 1) gene encodes a mitoNEET protein involved in mitochondrial labile iron and reactive oxygen species (ROS) homeostasis, contributing to the mitochondrial function and morphology^37,38^. Mitochondria dysfunction has been implicated in VSMC phenotypic switching and various cardiovascular diseases^39–41^. The exon 1 and exon 2 of *CISD1-u* transcript are the same as the annotated transcript *ENST00000488388*, and the exon 3 and exon 4 are the same as the exon 2 and exon 3 as the annotated transcript *ENST00000333926* (Supplementary Fig. 5e). Transcript *ENST00000333926* is the primary coding isoform of gene *CISD1*. The *CISD1-u* transcript was first validated by the 5’ RACE assay in HASMCs (Fig. 5a and Supplementary Fig. 7a). The junction and sequence of transcript *CISD1-u* were further verified by Sanger sequencing (Fig. 5b). To evaluate the function of *CISD1-u* in HASMC phenotypic switching, *CISD1-u* knockdown was performed and knockdown efficiency of *CISD1-u* was quantified on mRNA level (Fig. 5c, Supplementary Fig. 7b, Supplementary Fig. 6, and Supplementary Fig. 7c, d). *CISD1-u* knockdown showed no effect on the protein expression of primary *CISD1* (Fig. 5d). Upon the treatment of PDGF, HASMCs exhibited an increasing proliferation phenotype, which was characterized by increased expression of cycling genes (*CyclinD1* and *OPN1*) (Fig. 5e) and decreased expression of contraction genes (*ACTA2* and *TAGLN*) (Fig. 5f). The *CISD1-u* knockdown significantly increased the expression level of contraction genes and decreased the expression level of constriction genes in HASMCs with or without PDGF treatment (Fig. 5e, f). Consistently, the protein analyses confirmed the decreased CyclinD1 and elevated SMα-actin in PDGF-treated HASMCs upon *CISD1-u* knockdown (Fig. 5g, Supplementary Fig. 7e–g, and **5h**). In addition, cell proliferation and wound-healing assays showed that *CISD1-u* knockdown significantly inhibited the proliferative and wound-healing ability of HASMCs with or without PDGF treatment (Fig. 5i–k). Collectively, the unannotated transcript *CISD1-u* plays a striking role in the pathological phenotypic switch of HASMCs.

## Discussion

To investigate the roles of alternative splicing in phenotypic switch of human VSMCs, we used HASMCs treated with PDGF, TGFβ, and miR-221. In our analysis, the vast majority (76.36%) of detected transcripts were unannotated. The proportion of unannotated transcripts was comparable with previous studies using long-read RNA-seq data^28,42^. The major reason that led to the large portion of unannotated transcripts might be the transcript identification of long-read RNA-seq reads that could precisely characterize full-length transcripts, which largely improves read-to-transcript assignment and de novo transcript assembly that generates more unannotated transcript. The assembly and quantification of short-read RNA-seq deeply rely on the reference transcript and gene annotations. Long-read RNA-seq offers the opportunity to directly capture the full-length transcripts, and depends less on the transcript annotation. Thus, long-read RNA-seq could detect plenty of transcripts that were not annotated in the reference.

Due to various post-transcriptional regulation and modifications, including alternative splicing, different transcripts could be produced from the same genes in distinct conditions. Previous transcript-level studies have identified a number of specific RNA transcripts that were overlooked in gene-level analysis^22,23,28^. Long-read RNA-seq technology directly sequences full-length RNA transcripts, providing opportunities to precisely identify alternative splicing events and RNA transcripts^27^. In addition, we only considered splice junctions that are annotated in GENCODE (version 38), which removed the possibility to discover completely novel exons. Even though, our analysis identified a number of novel transcripts derived from known splice junctions and exons, which were more likely to be validly expressed than those from completely novel exons. In our dataset, nanopore long-read RNA-seq showed relatively lower coverage of known noncoding RNAs. One of the reasons is that poly(A) RNA-selected RNA libraries were used for nanopore RNA-seq, and only some portion of polyadenylated noncoding RNAs^43^ were detected. The other reason might be that the sequencing coverage of nanopore long-read RNA-seq is currently low, while most noncoding RNAs are expressed in low levels. We also identified dysregulated transcripts that their host genes showed no or reverse changes. These results highlighted the necessity of transcriptome analysis at the RNA transcript level, which is consistent with previous studies^23,28^. One of the limitations of ONT sequencing is the relatively high error rates of sequencing reads. This intrinsic defect could be attenuated by optimized base calling computational methods and correction with paired Illumina short-read data^44^, which were performed in our study. Another potential limitation is that a portion of transcripts were not captured in full length, especially those ultra-long transcripts. The assembly of novel transcripts would be biased around the ends.

Phenotypic transformation of VSMCs, dedifferentiated from a contractile phenotype to a migratory constriction phenotype, accounts for the pathogenesis of a variety of vascular diseases^45,46^. Various pathological stimuli such as circulating plasma substances, growth factors, inflammatory factors, and non-coding RNAs are involved in the complex network of VSMC phenotypic transition^47^. Here we chose three typical stimuli to perform ONT long-read RNA-seq analysis to uncover more accurate alternative splicing events: TGF-β stimulated contractile phenotypic model, PDGF-induced constriction phenotype, as well as miR-221 treatment, a non-coding RNA modulator in VSMC phenotype^14^, which is also a downstream target of PGDF. As expected, the percentage of transcriptional variations exclusively induced by TGFβ was much higher than PDGF and miR-221 treatment, accounts for 73% of upregulated transcripts in TGFβ group. Unexpectedly, in PDGF-treated HPASMC, highly expressed genes were enriched in “IL-17 signaling pathway”. Single-cell sequencing and lineage-tracing strategy have confirmed the dedifferentiated state of contractile VSMCs are prone to differentiate into macrophage-like VSMCs during atherogenesis, characterized with macrophage surface markers and functions^32,33^, but little is known about the Th17-related VSMCs phenotype. Further investigation is warranted to evaluate the clue by various annotated transcripts.

CISD1 belongs to the member of mitoNEET protein, containing an integral transmembrane region and a CDGSH domain^48,49^. The first 32 amino acids are necessary for its mitochondrial localization and the CDGSH domain (residues 55–93), oriented toward the cytoplasm, contributes to the iron transportation^50^. CISD1 has been implicated in mitochondrial bioenergetics, lipid metabolism, mitophagy, and inflammation^51^. Downregulating or deleting any one of the NEET proteins, including CISD1, leads to disruptions of mitochondrial function and morphology, as well as the metabolism and cellular bioenergetics^52,53^, which all play important roles in VSMC phenotypic switching^41^. Here we identified an unannotated *CISD1* transcript, *CISD1-u*, in HASMCs and its expression was significantly upregulated upon PDGF treatment. Interestingly, when the *CISD1-u* was deleted, HASMCs were converted to a differentiated phenotype, with lower proliferation potency. *CISD1-u* was a truncated transcript from known *CISD1*, including an inserted specific sequence. Considering the key role of CDGSH domain, the elevated *CISD-u* with deficit CDGSH domain may affect the mitochondrial homeostasis and the signaling communication between the mitochondria and cytoplasm. Then targeting the unannotated *CISD1-u* might be a promising therapy for VSMC phenotypic switching and related cardiovascular diseases.

## Methods

### Cell culture, treatment, and transfection

The primary human (male, 35 years old) aortic smooth muscle cells (HASMCs) were purchased from ScienCell Research Laboratories (Catalog #6110) and were cultured in smooth muscle cell specialty medium (Catalog #1101, ScienCell, USA). Cultured HASMCs were used between passages 3 and 6 supplemented with TGFβ (10 ng/ml) or PDGF (15 ng/ml) in a 5% CO_2_ incubator at 37 °C. Transfection of hsa-miR-221-3P (MIMAT0000278, AGCUACAUUGUCUGCUGGGUUUC) into the cells was carried out using the LipofectamineTM 2000 reagent (Invitrogen) according to the manufacturer’s protocol and at a final concentration of 50 nM. To maintain a similar cellular stress, control HASMCs and those with PDGF or TGFβ treatment were also transfected with control lipofectamine. Total RNA samples were prepared by using the TRIzol reagent (Invitrogen) according to the manufacturer’s instructions.

### RNA extraction, library construction, nanopore long-read RNA sequencing, and Illumina short-read RNA sequencing

Poly(A) RNA-selected total RNAs were extracted by using the NEBNext Poly(A) mRNA Magnetic Isolation Module according to the manufacturer’s instructions. For ONT long-read RNA-seq, the cDNA was synthesized following the strand-switching protocol of Oxford Nanopore Technologies. Briefly, the cDNA-PCR Sequencing kit by Oxford Nanopore (SQK-PCS109) was used to prepare full-length cDNA libraries from the poly(A) mRNAs. Then the cDNA was amplified by PCR for 13–14 cycles with specific barcoded adapters from the Oxford Nanopore PCR Barcoding kit (SQKPBK004). Finally, the 1D sequencing adapter was ligated to the cDNA before being loaded onto a FLOPRO002 R9.4.1 flow cell in a PromethION sequencer. MinKNOW was used to run the sequencing. For the Illumina short-read RNA-seq, a TruSeq kit (Illumina) was used to treat 1 μg of poly(A)-selected total RNAs with DNase I for library preparation. Prepared RNA samples were sequenced on an Illumina HiSeq X Ten machine. The library construction and sequencing of ONT long-read RNA-seq and Illumina short-read RNA-seq were both performed by Wuhan Benagen Technology Co., Ltd. (Wuhan, China). The raw sequencing data were deposited in the Gene Expression Omnibus (GEO) database under the accession number of GSE209739.

### Analysis of nanopore long-read RNA-seq data

The raw ONT long-read RNA-seq reads were first subject to the Porechop^54^ (version 0.2.4, https://github.com/rrwick/Porechop) tool to find and remove adapters. Then trimmed reads were processed following the FLAIR pipeline (version 1.4)^55^. In particular, minimap2^56^ (version 2.17-r941) was employed to align trimmed reads to the human reference genome (GRCh38) with the following parameters: *minimap2 -ax splice./reference/ref-human-ont.mmi $i/out.pass.fq -t 10 -o $i/aln.sam*. Indels were removed from the read alignments. The correct function implemented in FLAIR was utilized to correct the boundaries of splicing sites around reads. Only splice junctions that were annotated in the GENCODE^57^ v38 comprehensive gene annotation and covered by at least three uniquely mapped reads were considered valid in the following analysis. Unmatched splicing sites were corrected with the nearest valid splicing sites within 10 nt. Thus, only valid splicing sites were included in the corrected reads.

### Processing Illumina short-read RNA-seq data

The raw short-read RNA sequencing reads were first trimmed to remove adapters and low-quality bases by using Trimmomatic^58^ (version 0.39). All filtered reads were then aligned to the human reference genome (GRCh38) by HISAT2^59^ (version 2.2.1). This mapping strategy adopted possible splicing junctions in all samples. Final read alignments were subject to the StringTie^60^ (version 2.1.8) software for reference-based transcript assembly. Gene annotation of GENCODE v38 was utilized as the transcript model reference to guide the assembly in each sample. Finally, the merge mode implemented in StringTie was run to merge transcripts identified in all samples to produce a nonredundant master set of transcripts.

### Quantification of transcripts in nanopore and Illumina RNA-seq data

The quantify function implemented in FLAIR software was adopted to quantify transcripts identified in nanopore RNA-seq data, which only considered reads with alignment scores no less than 1. Transcripts detected from Illumina RNA-seq data were quantified by using StringTie (version 2.1.8) with assembled transcript annotation. Then quantification was normalized in the unit of transcripts per million mapped reads (TPM). Transcripts that expressed no less than 0.1 TPM in at least one sample remained for downstream analysis. In addition, the DESeq2^61^ software (version 1.30.1) was employed to identify DETs between different groups. Transcripts with fold change > 1.5 and FDR < 0.05 were considered as DETs.

### Enrichment analysis of transcripts

Annotated transcripts were directly mapped to known genes according to the GENCODE (v38) annotation, while genes of unannotated transcripts were searched by genomic coordinates using the BEDTools software (version 2.29.2)^62^. Only protein-coding genes remained for enrichment analysis. Then the clusterProfiler R package (version 4.1.4)^63^ was employed to conduct enrichment analysis in pathways curated in the Kyoto Encyclopedia of Genes and Genomes (KEGG) database^64^. The Benjamini-Hochberg corrected p values were adopted as the enrichment significance.

### Identification of alternative splicing events

Normalized transcript expression matrix and gene annotation were subject to SUPPA2 (version 2.3)^65^ for the identification of alternative splicing events (ASEs). Specifically, the “generateEvent” function with “-f ioe” option was used to generate local ASEs from the gene annotation GTF file. The percent spliced-in (PSI) value was calculated for each ASE in every sample by using the “psiPerEvent” function implemented in SUPPA2. ASEs with PSI values less than 0.1 in all samples were not included in downstream analysis. Then the Wilcoxon signed-rank test was employed to identify differentially spliced ASEs between different sample groups.

### Detection of isoform switching events

Genome-wide isoform switching events were identified by using the IsoformSwitchAnalyzeR package (version 1.17.04)^66^ with default parameters. Changes in isoform usage were computed by comparing the miR-221, PDGF, and TGFβ with the control group, respectively. The genome-wide enrichment was assessed by counting isoform switches of specific alternative splicing types and comparing the number of gains and losses by using the Proportion test (prop.test function in R) and Fisher’s exact test (fisher.test function in R). For each pairwise comparison, the mean gene and isoform expression values were used to calculate the isoform fraction. A switching event with absolute difference in isoform fraction > 0.1 and adjusted p-value < 0.05 was considered significant. Functional consequences of isoform switching events were also predicted. Particularly, we used external analyses with CAPT, IUPred2, SignalP, and Pfam tools to predict the coding capabilities, protein structure stability, peptide signaling, and shifts in protein domain usage of significantly switching isoforms. These analyses were imported back into IsoformSwitchAnalyzeR to infer downstream biological consequences.

### SiRNA transfection and validation

The silencing of *CISD1-u* was performed with the specific siRNA 5′-AAAUGAGUCUAAACAUGUCCA-3′ (Genepharma). *CISD1-u* siRNA transfection was conducted according to the protocol of DNA & siRNA transfection Reagent (jetPRIME). Cells were incubated in PDGF (10 ng/ml; MedChemExpress) for 24 h after siRNA transfection. Cellular protein and RNA were harvested after 24 h incubation in PDGF and the level of primary *CISD1* was quantified by western blot and *CISD1-u* silencing was analyzed by qPCR.

### Quantitative real-time PCR

Total RNA was isolated from HASMC cells with TRIzol reagent (Takara). The RNA was reverse-transcribed to cDNA using HiScript^®^ III All-in-one RT SuperMix Perfect for qPCR (Vazyme). Quantitative RT-PCR (qRT-PCR) was performed using ChamQ SYBR Color qPCR Master Mix (Vazyme) according to the manufacturer’s instructions. The gene expression was then detected by using the QuantStudio™ 7 Flex Real-Time PCR system (Applied Biosystems). Primer sequences were synthesized by GENEWIZ as follows. All data were quantified using the Comparative Cт method. qRT-PCR primers used in this study is provided in Supplementary Table 3.

### Western blotting

HASMC cells were lysed in RIPA buffer (Beyotime Biotechnology, Shanghai, China) in the presence of phenylmethylsulfonyl fluoride (PMSF, Beyotime). Protein content was determined by the BCA Protein Assay (Beyotime Biotechnology, Shanghai, China). The lysate was loaded in SDS-polyacrylamide gel and then subject to electrophoresis and electrical transfer. For CISD1, Cyclin D1, SMα-actin and GAPDH protein detection, rabbit anti-human CISD1 (ABclonal, A10317, 1:1000), rabbit anti-human Cyclin D1 (ABclonal, A11022, 1:1000), rabbit anti-human SMα-actin (Sigma-Aldrich, A2547, 1:500) and mouse anti-human GAPDH (ABclonal, AC002, 1:10000) antibodies were used, respectively. HRP Goat Anti-Rabbit IgG (Abclonal, AS014, 1:10000) and HRP Goat Anti-Mouse IgG (Abclonal, AS003, 1:5000) were used as secondary antibodies. In each lane, 15 μg protein was loaded. Detection of protein expression was performed by using Immobilon Western HRP (Millipore, MA, USA).

### 5′ RACE assays

Total RNAs were isolated from the HASMCs using RNAiso plus kit (Takara). RNA samples were subject to 5′-RACE reaction using the HiScript-TS 5′/3′RACE Kit (Vazyme, Nanjing, China). Firstly, the first-strand cDNA was synthesized according to the manufacturer’s instructions. Secondly, the synthesized cDNA was subject to a 5’-RACE reaction using forward and reverse primers: forward primers were the Universal Primer Mix, while reverse primer was a specific primer (5′-GCCCCATCACAGAATGGGAA-3′) for the novel transcript *CISD1-u*. Finally, the 5′-RACE product was subject to agarose gel electrophoresis, special bands extraction, and then confirmed by Sanger sequencing (Genewiz) using the following primers: upstream (5′-AGCATCGCGGAGTCGGT-3′) and downstream primer (5′-GCCCCATCACAGAATGGGAA-3′).

### Semiquantitative RT-PCR

To determine the percent splicing inclusion of novel transcript *CISD1-u*, total RNAs were isolated from HASMCs using RNAiso plus kit (Takara) and performed reverse transcription to generate cDNA according to the instruction of HiScript^®^ III All-in-one RT SuperMix Perfect for qPCR (Vazyme, Nanjing, China). The cDNA was then subject to a PCR reaction by following primers: GAPDH (F- GGACCTGACCTGCCGTCTAGAA; R-GGTGTCGCTGTTGAAGTCAGAG); *CISD1-u* (F-TGAGTTGTATGACGGCCACC; R-GCCCCATCACAGAATGGGAA); *CISD1* (F-ACCCGTTTGAGCTCGGTATC; R-TGTGAGCCCCATCACAGAAT). The PCR products were analyzed in 2% agarose gels containing Gel Red (Vazyme, Nanjing, China) and photographed under ultraviolet light. Finally, the grayscale values of PCR bands were analyzed using ImageJ software.

### Human aortic tissue collection

Aortic dissection tissues were collected during surgery from patients undergoing aortic root and ascending aorta replacement in the Department of Cardiac Surgery, Wuhan Asia Heart Hospital Affiliated to Wuhan University of Science and Technology. Control aortic tissues were collected from age-matched patients undergoing heart transplant surgery without aortic aneurysm, dissection or previous aortic repair. The study was reviewed and approved by the Ethics Committee of the Wuhan Asia Heart Hospital Affiliated to Wuhan University of Science and Technology and written informed consents were obtained from all patients.

### Proliferation activity (CCK8 assay)

Approximately 4 × 10^4^ primary HASMCs of passages 3 to 6 were plated in each well of a 96-well plate for 24 h. Cells were transfected with scramble or si-*CISD1-u* for another 36 h. Subsequently, vehicle control or PDGF (15 ng/mL) was added and incubated for 24 h. 10× diluted CCK8 solution (Solarbio, Beijing, China) was added to the culture medium and incubated for 2 h. The absorbance of the solution was measured at 450 nm using a MRX II absorbance reader (Dynex Technologies, Chantilly, VA).

### Migration activity (scrape assay)

Primary HASMCs of passages 3 to 6 were seeded in 24-well plates at a concentration of 1 × 10^5^ cells/well and transfected with scramble or si-*CISD1-u* for 36 h. A linear wound was gently introduced in the center of the cell monolayer using a 200-µl tip followed by washing away the cellular debris with PBS. Thereafter, cells were stimulated with or without PDGF (15 ng/mL) and incubated for another 24 h. The borders of the scrape were outlined on the bottom of the plate, images were acquired, and the area of migration was measured by automated planimetry using ImageJ software (National Institutes of Health).

### Statistics and reproducibility

Statistical analysis and data visualization in this study were performed by using the R software (R Foundation for Statistical Computing, Vienna, Austria; http://www.r-project.org) and the GraphPad Prism 8.0 software. Normality was examined using the Shapiro-Wilk test. Two-tailed unpaired Student’s t-test was performed to compare two datasets. Multiple comparisons were tested using one or two-way analysis of variance (ANOVA) followed by Bonferroni’s post-test. Unless specific statements, all tests were performed in two-sided, and *p* or FDR values < 0.05 were considered statistically significant.

### Reporting summary

Further information on research design is available in the Nature Portfolio Reporting Summary linked to this article.

### Supplementary information

Supplementary information

Description of Additional Supplementary Files

Supplementary Data 1

Supplementary Data 2

Supplementary Data 3

Supplementary Data 4

Supplementary Data 5

Supplementary Data 6

Supplementary Data 7

Supplementary Data 8

reporting summary

## Data Availability

The raw nanopore long-read RNA-seq and Illumina short-read RNA-seq data generated in this study was deposited in the GEO database with the accession number of GSE209739. The alignment bam files of nanopore long-read and Illumina short-read RNA-seq data were deposited in the Sequence Read Archive (SRA) database with the accession of PRJNA1001518. Software and resources used for analysis and visualization are described in each method section. All results generated in this study can be found in supplemental tables. Source data for all figures except for Fig. 4a are provided in Supplementary Data 7. Source data underlying Fig. 4a are provided in Supplementary Data 8.

## Code availability

Scripts and codes that were used for data analysis and visualization were deposited in GitHub^67^: https://github.com/lishenglilab/ONT-LR-seq-in-HVSMC.
