# Supplementary material for: Nanopore long-read RNA sequencing reveals functional alternative splicing variants in human vascular smooth muscle cells
Source: Commun Biol. 2023 Oct 31;6:1104. doi: 10.1038/s42003-023-05481-y (PMC10618188; doi:10.1038/s42003-023-05481-y)
Supplement: Supplementary file 1 — Supplementary information [file 42003_2023_5481_MOESM1_ESM.pdf]

Supplementary Tables

Supplementary Table 1. Statistics of nanopore long-read RNA sequencing.

Sample	Data (GB)	Total reads	Pass (%)	Median pass read length	Mean pass read length	Mapped (%)	Gene No.	Transcript No.	No. of Gene with AS	Gene with AS (%)
Control_rep1	8.86	10,468,537	96.72	1,296	924	78.66	13,164	223,412	10,540	80.07
Control_rep2	8.77	10,569,236	96.53	1,281	905	77.35	13,340	235,453	10,589	79.38
Control_rep3	9.15	10,391,495	96.96	1,344	970	79.9	13,288	228,053	10,608	79.83
Control_rep4	9.48	10,526,110	96.79	1,387	970	80.01	13,319	238,223	10,585	79.47
PDGF_rep1	10.23	11,676,047	97.48	1,445	990	77.99	13,299	247,104	10,594	79.66
PDGF_rep2	10.24	12,478,598	97.36	1,366	960	75.67	13,312	244,556	10,566	79.37
PDGF_rep3	12.26	14,056,850	97.56	1,387	900	76.36	13,478	262,739	10,660	79.09
PDGF_rep4	11.24	11,885,063	97.54	1,504	917	79.7	13,383	260,137	10,628	79.41
TGFβ_rep1	12.24	12,887,787	97.66	1,470	1,013	76.14	13,539	266,938	10,666	78.78
TGFβ_rep2	11.21	11,325,150	97.74	1,510	975	76.11	13,341	250,289	10,618	79.59
TGFβ_rep3	9.77	10,727,962	98.11	1,536	1,003	75	13,239	240,514	10,573	79.86
TGFβ_rep4	11.18	12,844,843	98.20	1,484	995	75.89	13,441	264,406	10,620	79.01
miR-221_rep1	11.16	11,836,846	98.35	1,513	1,002	75.08	13,438	256,315	10,627	79.08
miR-221_rep2	12.24	13,733,383	97.83	1,480	960	75.68	13,599	282,628	10,695	78.65
miR-221_rep3	12.26	13,176,564	97.47	1,449	939	74.79	13,473	268,187	10,659	79.11
miR-221_rep4	12.23	13,254,937	97.93	1,555	1,002	74.87	13,534	281,835	10,683	78.93

Supplementary Table 2. The numbers of mapped reads of ONT and Illumina RNA-seq platform in each sample.

Sample	ONT (million)	Illumina (million)
Control_rep1	6.64	93.99
Control_rep2	6.82	93.46
Control_rep3	6.94	100.70
Control_rep4	7.09	96.81
PDGF_rep1	7.67	102.01
PDGF_rep2	7.87	88.78
PDGF_rep3	8.91	107.20
PDGF_rep4	8.01	105.36
TGFβ_rep1	8.28	112.30
TGFβ_rep2	7.35	100.60
TGFβ_rep3	6.85	109.04
TGFβ_rep4	8.05	99.62
miR-221_rep1	7.53	103.75
miR-221_rep2	8.81	96.03
miR-221_rep3	8.28	100.48
miR-221_rep4	8.56	100.46

Supplementary Table 3. qRT-PCR primers used in this study.

Gene name	Forward	Reverse
FOXS1-u	5′-CCAGGCTGGTTAAAAGGTCCA-3′	5′-TCAGCAGACCCACCCCTTTT-3′
CACNA2D3-u	5′- TCGATCCCATTCAGCACTGG-3′	5′- AGCATTTGCCATCCAGGGAA-3′
ADAM19-u	5′-AGGCTGGTCATTGACTTCGG-3′	5′-CCCTGCTCTGACTGTTCCAG-3′
MACF1-u	5′-GTTCTGCAAATCAGCTTCAGCA-3′	5′- GCAAAGCTGCCTTCATACCAT-3′
MAP2-u	5′-AGGGCTGGTAGGTTGGATCT-3′	5′-TGGGTCACCAAAAAGCACCT-3′
CISD1-u	5′-TGAGTTGTATGACGGCCACC-3′	5′-GCCCCATCACAGAATGGGAA-3′
RFX8-u	5′-CCTCGCCCAGACCAATTCAT-3′	5′-ACTTGCTTGAGCGTTGGAGT-3′
Cyclin D1	5′-AGAGGCGGAGGAGAACA-3′	5′-GAGAGGAAGCGTGTGAGG-3′
OPN	5′-CTCCATTGACTCGAACGAC-3′	5′-GTGAAAACTTCGGTTGCTG-3′
SMα-actin	5′-CGTGGCTATTCCTTCGTT-3′	5′-ACGTTCATTTCCGATGGT-3′
SM22α	5′-GGCTGAAGAATGGCGTGATT-3′	5′-TCTGCTTGAAGACCATGGAGG-3′
CISD1	5′-ACCCGTTTGAGCTCGGTATC-3′	5′-TGTGAGCCCCATCACAGAAT-3′
TAGLN	5′-ATGACAGGCTACGGACGAC-3′	5′-GTGGGTGAGGCAGGCTA-3′
CNN1	5′-CATCCTCCAGCCCCTGT-3′	5′-GTTGCTCAGTGCGTCCTTT-3′
TCIM-U	5′-TGTAACCCTCCCAGCAGATG-3′	5′-GGGTTTAGAGTCTGAGTGATGA-3′
HSPH1-U	5′-ACTGGATGTCGTCATGGTGG-3′	5′-CCACTGTAATTCCCTGTGTGC-3′
MDM2-U	5′-ATTGTGGGTTTAGGTTGCTGT-3′	5′-ACCCCAGTATTCGTTGATGTC-3′
ITM2B-U	5′-GTTGGCCAAAGAAGAGCCTG-3′	5′-TACCACTCTGGCTGAAGGTC-3′
FAM114A1-U	5′-AGATGCAGCGCCTTCTTTAG-3′	5′-GTCAGGAGAGTCCTAAGCTGC-3′
KIT	5′-GGATCACGGAAAAGGCAGAA-3′	5′-GAGCGGTCAACAAGGAAAAGC-3′
CDKN1B	5′-CCGAGGTGCTTGGGAGTTTT-3′	5′-TGTTTACACAGCCCGAAGTG-3′
MYOCD	5′-TCCTGTGGATTCTGCTGTGAAA-3′	5′-TGAGTTTTGGGGGTCTTCACTT-3′
GAPDH	5′-GGACCTGACCTGCCGTCTAGAA-3′	5′-GGTGTCGCTGTTGAAGTCAGAG-3′

Supplementary Figures

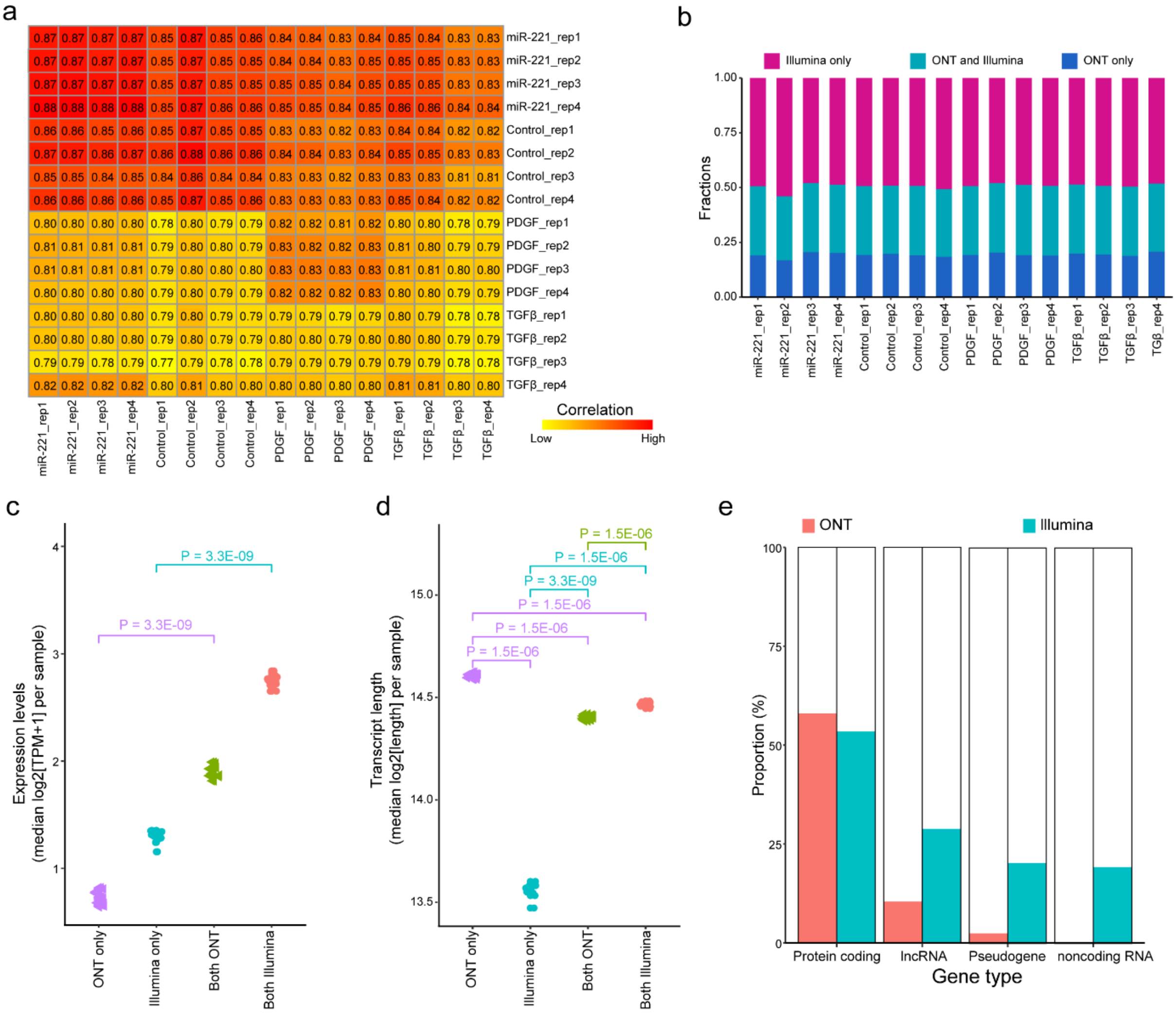

Supplementary Figure 1. Comparisons of sequencing data between ONT and Illumina platforms. a Correlations of transcript expression between ONT RNA-seq and Illumina RNA-seq. **b** Stack plot shows the fractions of transcripts detected by different sequencing technologies in each sample. **c** Comparisons of expression levels among transcripts that were detected by ONT only, Illumina only, and both. P, Wilcoxon's rank-sum test. **d** Comparisons of length among transcripts that were detected by ONT only, Illumina only, and both. P, Wilcoxon's rank-sum test. **e** The proportion of known genes detected by ONT and Illumina in different gene types.

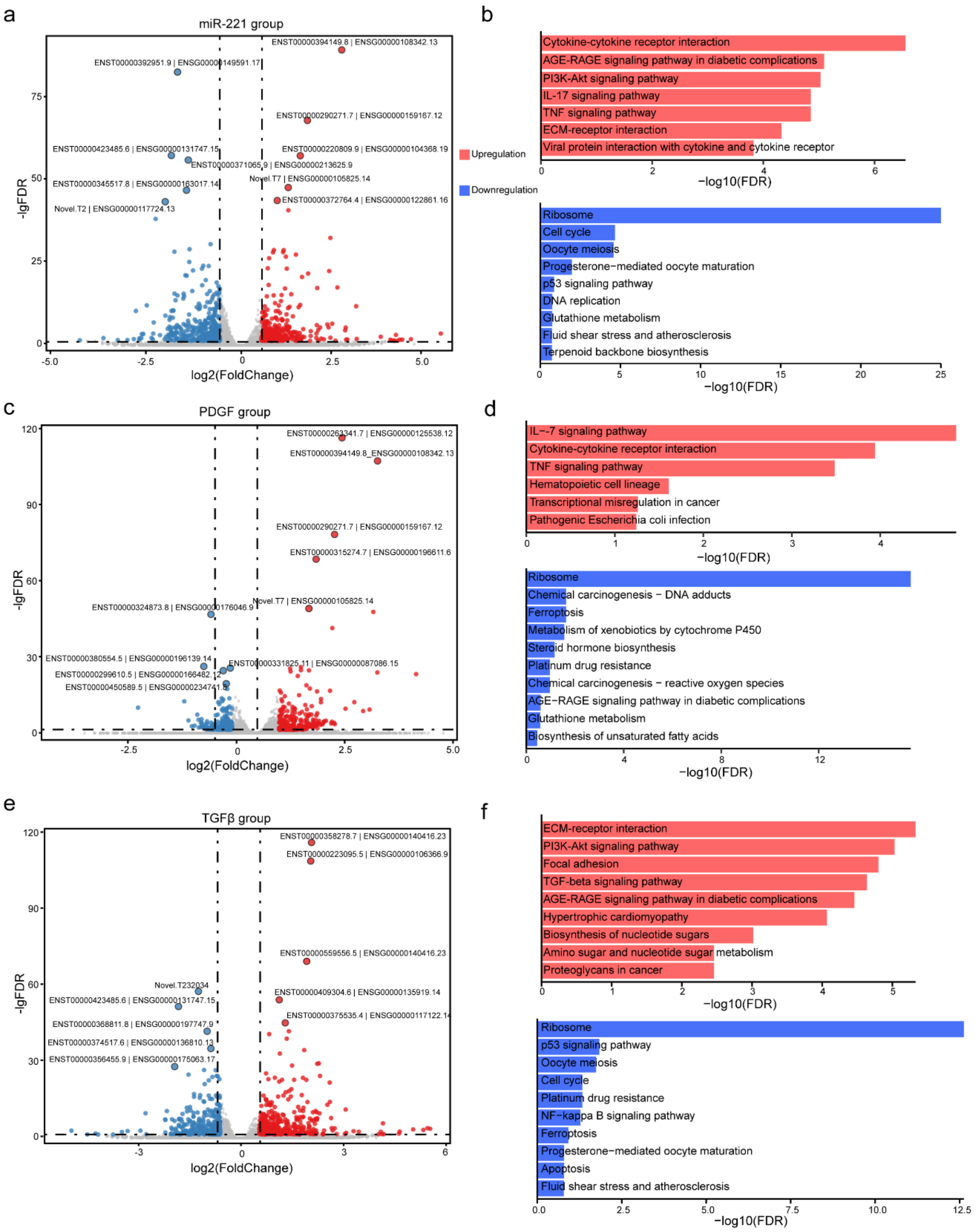

Supplementary Figure 2. Differential transcripts in the miR-221, PDGF, and TGFβ groups. a Volcano plot shows the differential significance of transcripts in the miR-221 group. **b** Significantly enriched KEGG pathways by upregulated and downregulated transcripts in the miR-221 group. **c** Volcano plot shows the differential significance of transcripts in the PDGF group. **d** Significantly enriched KEGG pathways by upregulated and downregulated transcripts in the PDGF group. **e** Volcano plot shows the differential significance of transcripts in the TGFβ group. **f** Significantly enriched KEGG pathways by upregulated and downregulated transcripts in the TGFβ group.

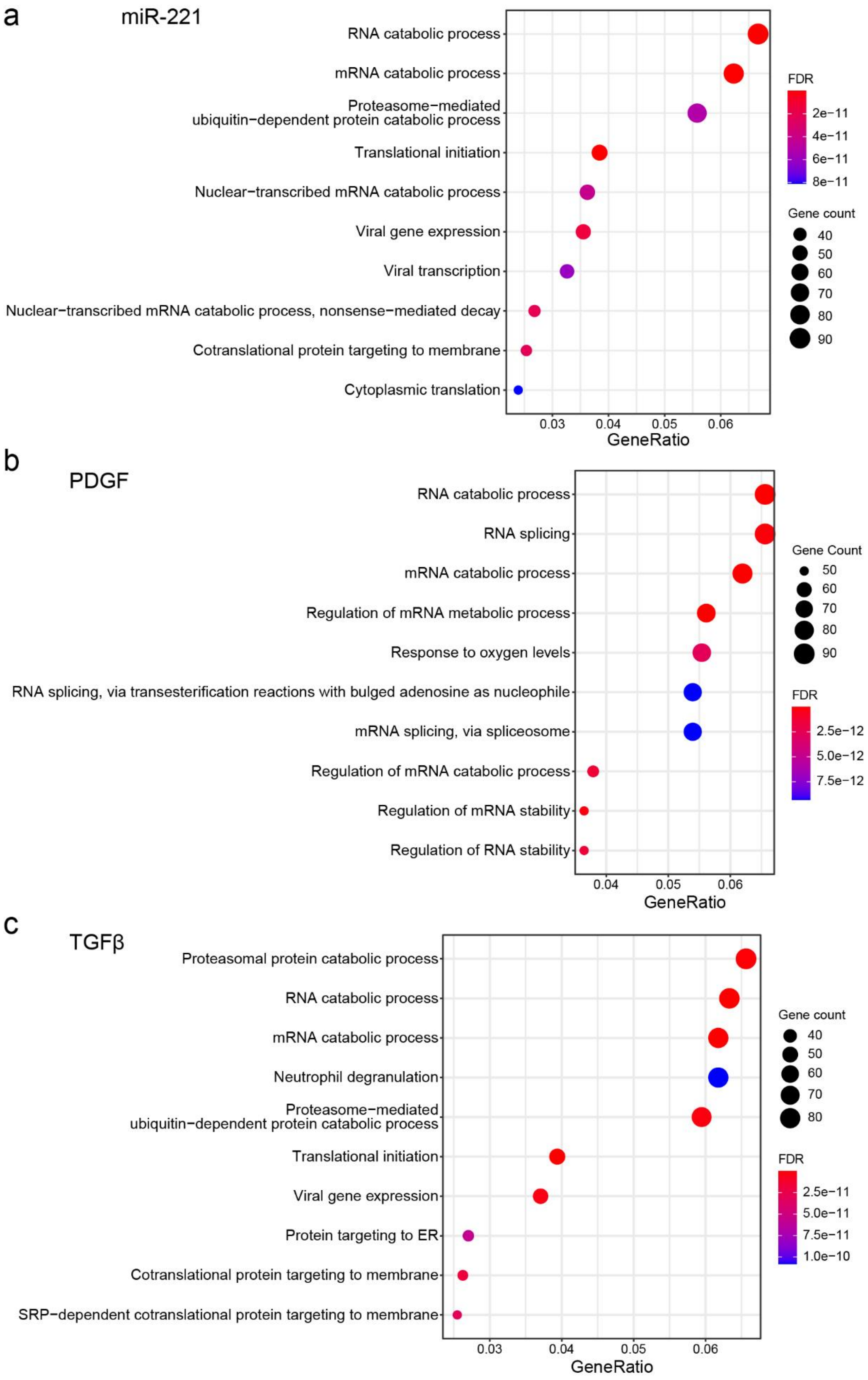

Supplementary Figure 3. Enrichment analysis in different groups. GO biological processes enrichment of differentially spliced genes in the miR-22 (a), PDGF (b), and TGFβ (c) HASMC samples.

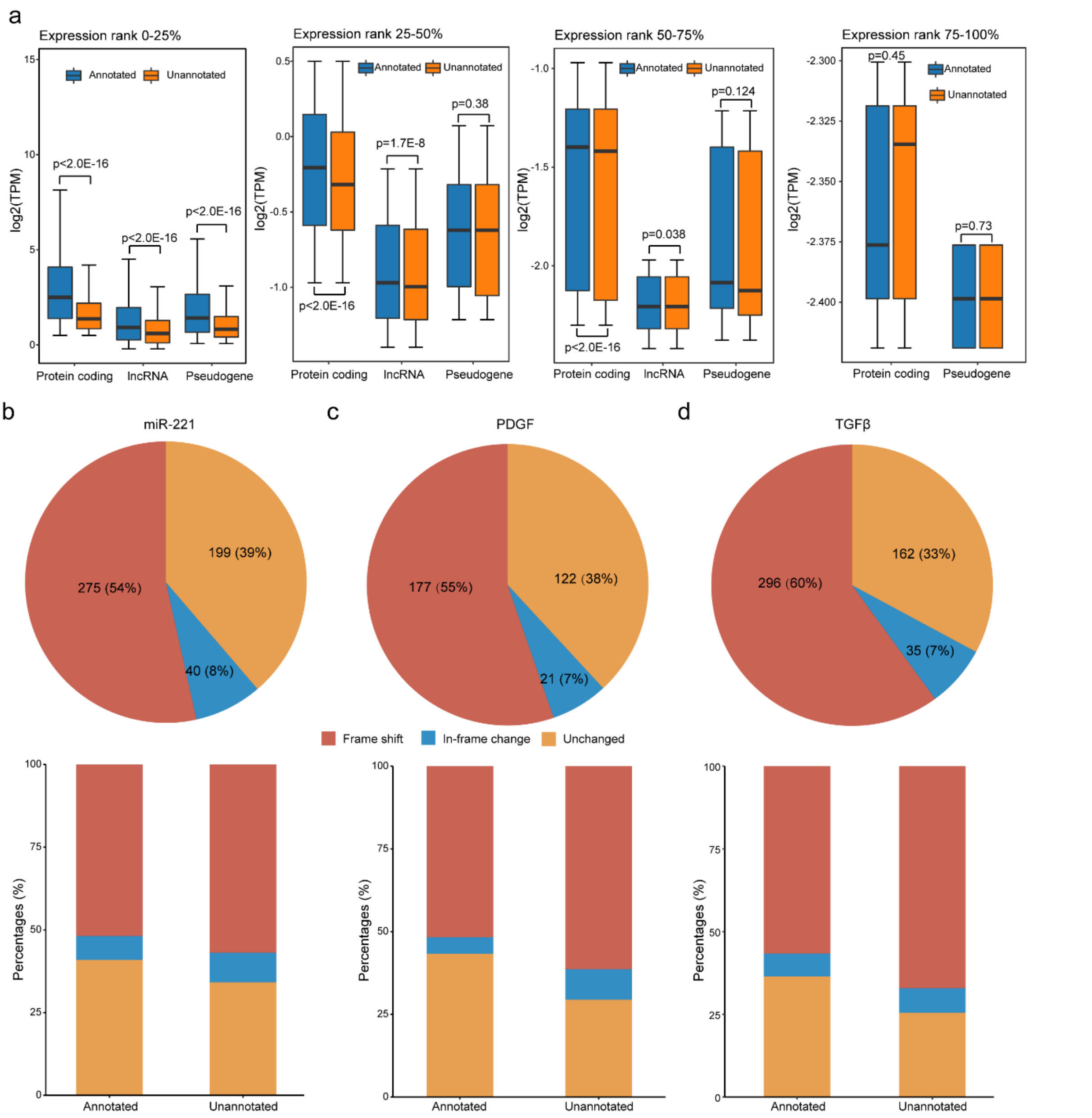

Supplementary Figure 4. The expression levels and ORF changes of unannotated transcripts. a Comparisons of expression levels between annotated and unannotated transcripts in different ranges of the expression rank. Each box represents the IQR and median of TPM value of each transcript, whiskers indicate 1.5 times the IQR. P, Wilcoxon's rank-sum test. The percentage of ORF changes in the miR-221 group (**b**), PDGF group (**c**), and TGFβ group (**d**). The bar plots below each pie chart are the percentages of ORF changes in annotated and unannotated transcripts.

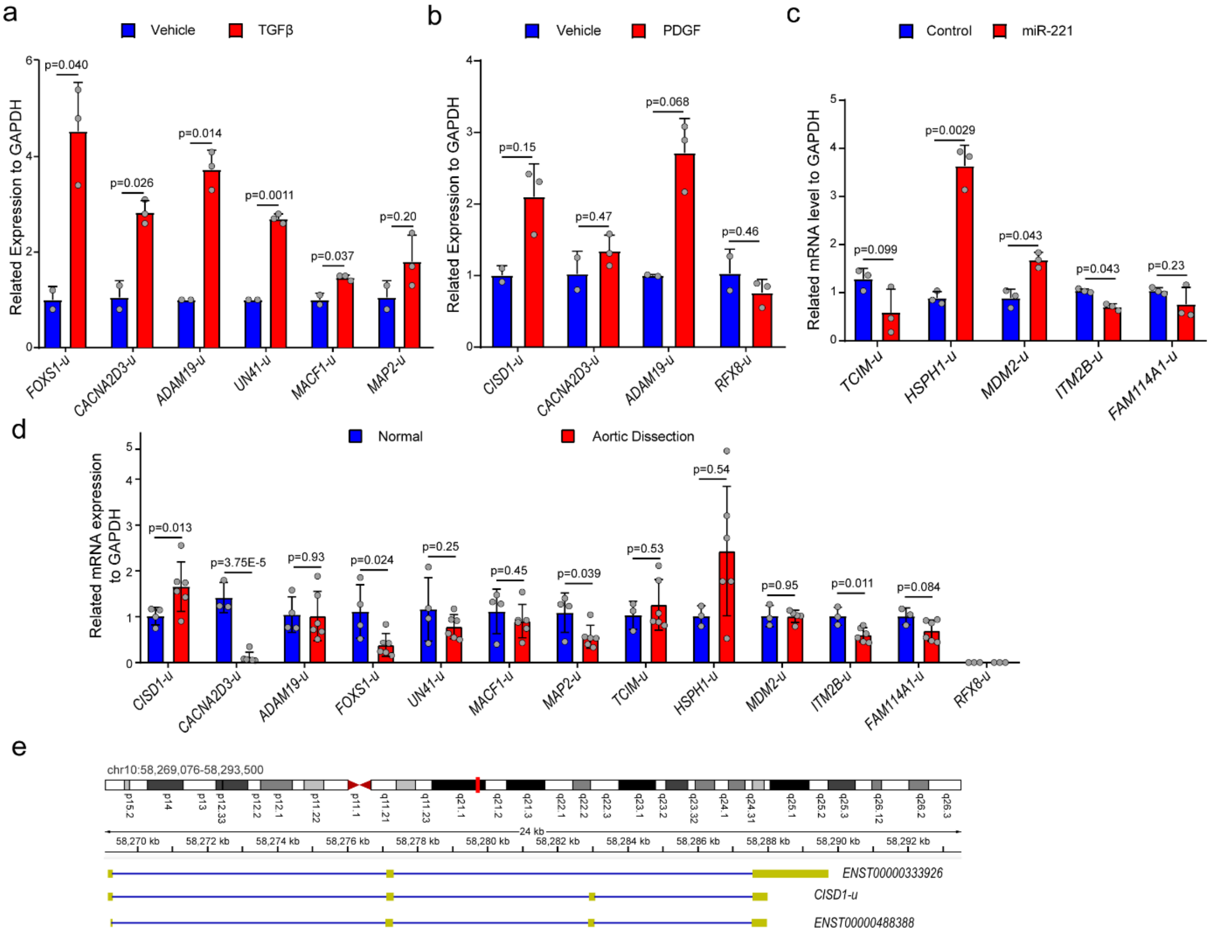

Supplementary Figure 5. The validation of unannotated transcripts. a qRT-PCR validation of selected novel transcripts in TGFβ-treated primary HASMCs (10 ng/ml, 24 h). **b** qRT-PCR validation of selected novel transcripts in PDGF-treated primary HASMCs (15 ng/ml, 24 h). **c** qRT-PCR validation of selected novel transcripts in negative control miRNA or hsa-miR-221-3P-transfected primary HASMCs (50 nM, 60 h). **d** qRT-PCR validation of selected novel transcripts in human aortic vascular tissues. **e** The genomic coordinates (GRCh38) of transcript *ENST00000333926.6*, *CISD1-u*, and *ENST00000464703.5*. Three to six independent samples were used in each group. Error bars represent the means ± SDs. P, unpaired Student's t test.

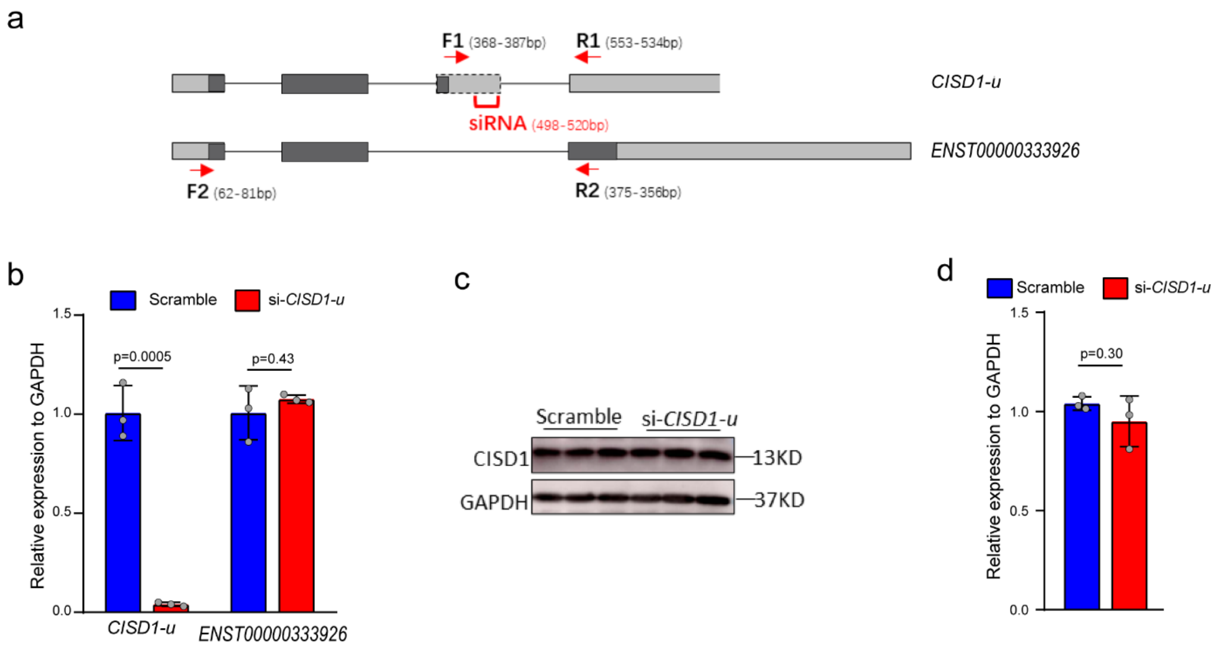

Supplementary Figure 6. The knockdown effect of *CISD1-u* transcript on both isoforms in HASMCs. a Schematic of experimental design for primers of both transcripts and siRNA for novel transcript *CISD1-u*. Dark gray indicates coding regions. **b** The efficiency of novel transcript *CISD1-u* knockdown in HASMCs on mRNA level of both isoforms. Primary HASMCs were transfected with scramble or si-*CISD1-u* (50 nM) for 48h for mRNAs. Three independent samples were used in each group. **c** Western blot analysis of *CISD1-u* knockdown on the protein expression of CISD1. **d** Quantitative analysis of *CISD1-u* knockdown on the protein expression of CISD1. Primary HASMCs were transfected with scramble or si-*CISD1-u* (50 nM) for 72h for proteins. Three independent samples were used in each group. Error bars represent the means ± SDs. P, unpaired Student's t test

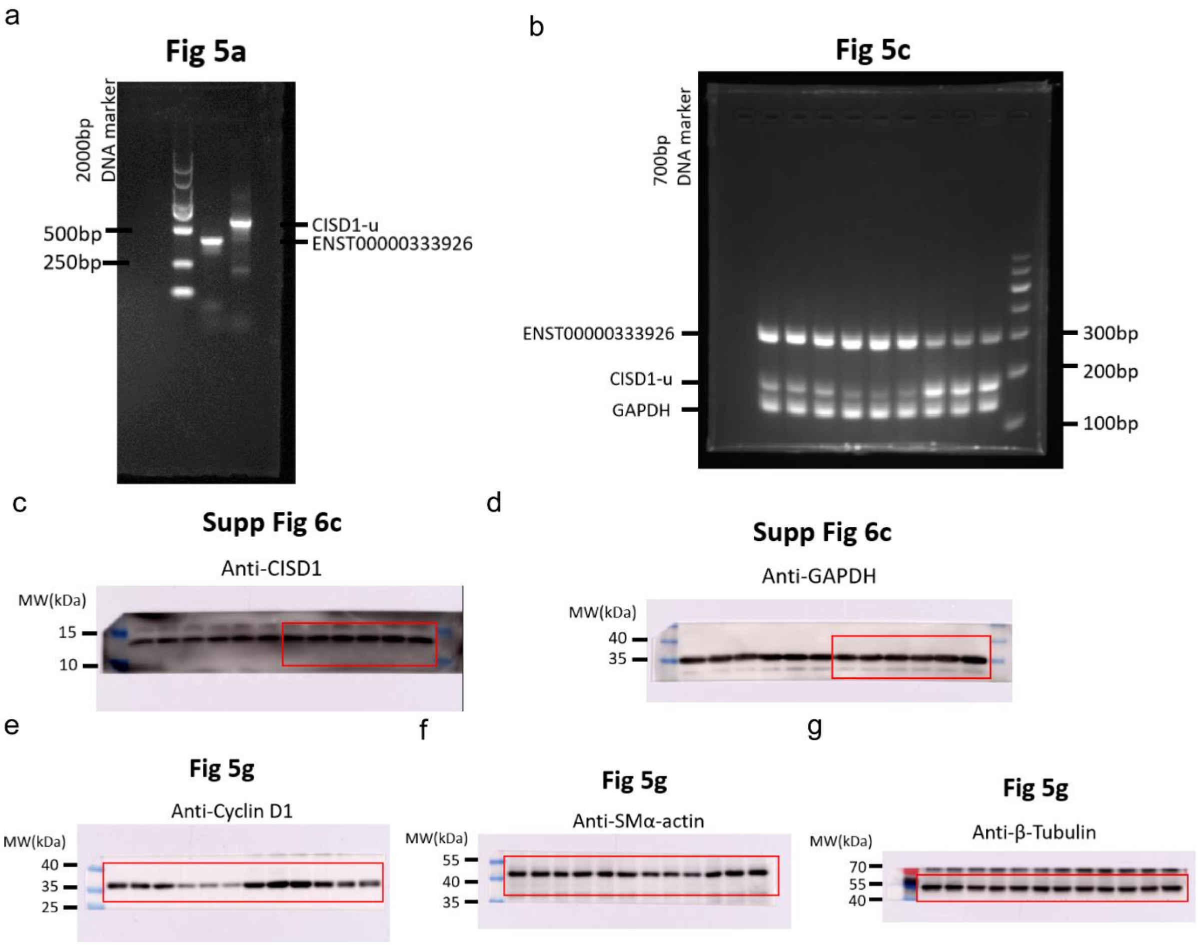

Supplementary Figure 7. Original gels for Fig. 5 and Supplementary Figure 6. (**a**) and (**b**) display the uncut agarose gels for Fig. 5a and Fig. 5c. (**c**) and (**d**) display the original western blot for Supplementary Figure 6c, which are derived from the same blot and was cut into strips for two proteins assay. (**e-g**) display the original western blot for Fig. 5g, which are derived from different runs with same sample loading.
