# Supplementary material for: Nanopore long-read RNA sequencing reveals functional alternative splicing variants in human vascular smooth muscle cells
Source: Commun Biol. 2023 Oct 31;6:1104. doi: 10.1038/s42003-023-05481-y (PMC10618188; doi:10.1038/s42003-023-05481-y)
Supplement: Supplementary file 2 — Description of Additional Supplementary Files [file 42003_2023_5481_MOESM2_ESM.pdf]

Description of Additional Supplementary Files

File name: Supplementary Data 1
Description: Differential transcripts analysis between normal and other groups

File name: Supplementary Data 2
Description: KEGG pathway enrichment analysis of differentially expressed transcripts in each group

File name: Supplementary Data 3
Description: The expression profile of differential transcripts in Fig. 2c

File name: Supplementary Data 4
Description: KEGG pathway enrichment analysis of differential transcripts in Fig. 2c

File name: Supplementary Data 5
Description: The PSI values of filtered alternative splicing events identified in each sample

File name: Supplementary Data 6
Description: The isoform switching events identified in each group

File name: Supplementary Data 7
Description: Source data for all files except Fig. 4a

File name: Supplementary Data 8
Description: Source data for Fig.4a
