# Supplementary material for: Nanopore long-read RNA sequencing reveals functional alternative splicing variants in human vascular smooth muscle cells
Source: Commun Biol. 2023 Oct 31;6:1104. doi: 10.1038/s42003-023-05481-y (PMC10618188; doi:10.1038/s42003-023-05481-y)
Supplement: Supplementary file 11 — reporting summary [file 42003_2023_5481_MOESM11_ESM.pdf]

nature portfolio

Corresponding author(s): Shengli Li

Last updated by author(s): Oct 13, 2023

Reporting Summary

Nature Portfolio wishes to improve the reproducibility of the work that we publish. This form provides structure for consistency and transparency in reporting. For further information on Nature Portfolio policies, see our Editorial Policies and the Editorial Policy Checklist.

Statistics

For all statistical analyses, confirm that the following items are present in the figure legend, table legend, main text, or Methods section.

n/a	Confirmed	
☐	☒	The exact sample size (*n*) for each experimental group/condition, given as a discrete number and unit of measurement
☐	☒	A statement on whether measurements were taken from distinct samples or whether the same sample was measured repeatedly
☐	☒	The statistical test(s) used AND whether they are one- or two-sided<br>*Only common tests should be described solely by name; describe more complex techniques in the Methods section.*
☐	☒	A description of all covariates tested
☐	☒	A description of any assumptions or corrections, such as tests of normality and adjustment for multiple comparisons
☐	☒	A full description of the statistical parameters including central tendency (e.g. means) or other basic estimates (e.g. regression coefficient) AND variation (e.g. standard deviation) or associated estimates of uncertainty (e.g. confidence intervals)
☐	☒	For null hypothesis testing, the test statistic (e.g. *F*, *t*, *r*) with confidence intervals, effect sizes, degrees of freedom and *P* value noted<br>*Give P values as exact values whenever suitable.*
☒	☐	For Bayesian analysis, information on the choice of priors and Markov chain Monte Carlo settings
☒	☐	For hierarchical and complex designs, identification of the appropriate level for tests and full reporting of outcomes
☐	☒	Estimates of effect sizes (e.g. Cohen's *d*, Pearson's *r*), indicating how they were calculated

Our web collection on statistics for biologists contains articles on many of the points above.

Software and code

Policy information about availability of computer code

Data collection	No software was used for data collection.
Data analysis	Porechop (version 0.2.4), Minimap2 (version 2.17-r941), FLAIR (version 1.4), Trimmomatic (version 0.39), HISAT2 (version 2.2.1), StringTie (version 2.1.8), DESeq2 R package (version 1.30.1), BEDTools (version 2.29.2), clusterProfiler R package (version 4.1.4), SUPPA2 (version 2.3), IsoformSwitchAnalyzeR package (version 1.17.04), GraphPad Prism 8.0

For manuscripts utilizing custom algorithms or software that are central to the research but not yet described in published literature, software must be made available to editors and reviewers. We strongly encourage code deposition in a community repository (e.g. GitHub). See the Nature Portfolio guidelines for submitting code & software for further information.

Data

Policy information about availability of data

All manuscripts must include a data availability statement. This statement should provide the following information, where applicable:

- Accession codes, unique identifiers, or web links for publicly available datasets
- A description of any restrictions on data availability
- For clinical datasets or third party data, please ensure that the statement adheres to our policy

The raw nanopore long-read RNA-seq and Illumina short-read RNA-seq data generated in this study was deposited in the GEO database with the accession number of GSE209739. The alignment bam files of nanopore long-read and Illumina short-read RNA-seq data were deposited in the Sequence Read Archive (SRA) database

with the accession of PRJNA1001518. Software and resources used for analysis and visualization are described in each method section. All results generated in this study could be found in supplemental tables.

Human research participants

Policy information about studies involving human research participants and Sex and Gender in Research.

Reporting on sex and gender	Not relevant in our study. No tissues derived from human were used in our study.
Population characteristics	Not relevant in our study.
Recruitment	Not relevant in our study.
Ethics oversight	Not relevant in our study.

Note that full information on the approval of the study protocol must also be provided in the manuscript.

Field-specific reporting

Please select the one below that is the best fit for your research. If you are not sure, read the appropriate sections before making your selection.

☒ Life sciences ☐ Behavioural & social sciences ☐ Ecological, evolutionary & environmental sciences

For a reference copy of the document with all sections, see nature.com/documents/nr-reporting-summary-flat.pdf

Life sciences study design

All studies must disclose on these points even when the disclosure is negative.

Sample size	16 human aortic smooth muscle cells (HASMC) samples were used for long-read RNA-seq.
Data exclusions	No data exclusion.
Replication	For long-read RNA-seq, these samples were derived from normal, PDGF-cultured, TGFβ-cultured, and hsa-miR-221-3P-transfected human VSMCs, with four replicate samples in each group; For experimental validation, three replicates were used in each experiment.
Randomization	Randomization is not relevant to our study.
Blinding	Blinding is not relevant to our study.

Reporting for specific materials, systems and methods

We require information from authors about some types of materials, experimental systems and methods used in many studies. Here, indicate whether each material, system or method listed is relevant to your study. If you are not sure if a list item applies to your research, read the appropriate section before selecting a response.

Materials & experimental systems

n/a	Involved in the study	
☐	☒	Antibodies
☒	☐	Eukaryotic cell lines
☒	☐	Palaeontology and archaeology
☒	☐	Animals and other organisms
☒	☐	Clinical data
☒	☐	Dual use research of concern

Methods

n/a	Involved in the study	
☒	☐	ChIP-seq
☒	☐	Flow cytometry
☒	☐	MRI-based neuroimaging

Antibodies

Antibodies used	For CISD1, Cyclin D1, α-SMA and GAPDH protein detection, rabbit anti-human CISD1 (ABclonal, A10317), rabbit anti-human Cyclin D1 (ABclonal, A11022), rabbit anti-human SMα-actin (Sigma-Aldrich, A2547) and mouse anti-human GAPDH (ABclonal,AC002) antibodies were used, respectively. HRP Goat Anti-Rabbit IgG (Abclonal, AS014) and HRP Goat Anti-Mouse IgG (Abclonal, AS003) were used as secondary antibodies.

Validation

Western blot analysis of extracts of various cell lines, using CISD1 antibody (A10317) at 1:1000 dilution. Secondary antibody: HRP Goat Anti-Rabbit IgG (H+L) (AS014) at 1:10000 dilution. Lysates/proteins: 25ug per lane; Blocking buffer: 3% nonfat dry milk in TBST; Detection: ECL Basic Kit (RM00020); Exposure time: 10s.
Western blot analysis of extracts of various cell lines, using Cyclin D1 antibody (A11022) at 1:1000 dilution. Secondary antibody: HRP Goat Anti-Rabbit IgG (H+L) (AS014) at 1:10000 dilution. Lysates/proteins: 25ug per lane;Blocking buffer: 3% nonfat dry milk in TBST;Detection: ECL Basic Kit (RM00020);Exposure time: 30s.
Western blot analysis of extracts of various cell lines, using GAPDH antibody (AC002) at 1:10000 dilution. Secondary antibody: HRP Goat Anti-Mouse IgG (H+L) (AS003) at 1:10000 dilution. Lysates/proteins: 25ug per lane; Blocking buffer: 3% nonfat dry milk in TBST; Detection: ECL Basic Kit (RM00020); Exposure time: 1s.
Western blot analysis of extracts of HeLa cells, using GAPDH (AC002) antibody as the primary antibody at dilution of 1:80000. Secondary antibody: using HRP Goat Anti-Rabbit IgG (H+L) antibody (AS014) at 1:4000-1:10000 dilution. Lysates/proteins: 25ug per lane; Blocking buffer: 3% nonfat dry milk in TBST; Detection: ECL Basic Kit (RM00020); Exposure time: 3s.
Western blot analysis of extracts of HeLa cells, using GAPDH antibody (ABclonal, AC002) as the primary antibody. Secondary antibody: HRP Goat Anti-Mouse IgG (H+L) antibody (AS003) at 1:5000/1:10000 dilution. Lysates/proteins: 25ug per lane; Blocking buffer: 3% nonfat dry milk in TBST; Detection: ECL Basic Kit (RM00020); Exposure time: 60s.
